# The Desmin (*DES*) Mutation p.A337P Is Associated with Left-Ventricular Non-Compaction Cardiomyopathy

**DOI:** 10.3390/genes12010121

**Published:** 2021-01-19

**Authors:** Olga Kulikova, Andreas Brodehl, Anna Kiseleva, Roman Myasnikov, Alexey Meshkov, Caroline Stanasiuk, Anna Gärtner, Mikhail Divashuk, Evgeniia Sotnikova, Sergey Koretskiy, Maria Kharlap, Viktoria Kozlova, Elena Mershina, Polina Pilus, Valentin Sinitsyn, Hendrik Milting, Sergey Boytsov, Oxana Drapkina

**Affiliations:** 1 National Medical Research Center for Therapy and Preventive Medicine, Petroverigsky per., 10, bld. 3, 101000 Moscow, Russia; olgakulikova2014@mail.ru (O.K.); andorom@yandex.ru (R.M.); meshkov@lipidclinic.ru (A.M.); divashuk@gmail.com (M.D.); sotnikova.evgeniya@gmail.com (E.S.); SKoretskiy@gnicpm.ru (S.K.); kharlapmaria@yahoo.com (M.K.); vkozlova2905@gmail.com (V.K.); drapkina@bk.ru (O.D.); 2 Erich and Hanna Klessmann Institute, Heart and Diabetes Center NRW, University Hospital of the Ruhr-University Bochum, Georgstrasse 11, 32545 Bad Oeynhausen, Germany; cstanasiuk@hdz-nrw.de (C.S.); agaertner@hdz-nrw.de (A.G.); hmilting@hdz-nrw.de (H.M.); 3 Kurchatov Genomics Center-ARRIAB, All-Russia Research Institute of Agricultural Biotechnology, Timiryazevskaya Street, 42, 127550 Moscow, Russia; 4 Medical Research and Educational Center, Lomonosov Moscow State University, Lomonosovsky Prospect 27, Building 10, 119991 Moscow, Russia; elena_mershina@mail.ru (E.M.); pilius.polina@botkin.ai (P.P.); vsini@mail.ru (V.S.); 5 National Medical Research Center for Cardiology, 3-ya Cherepkovskaya Street, 15A, 121552 Moscow, Russia; prof.boytsov@gmail.com

**Keywords:** cardiomyopathy, desmin, *DES*, desminopathy, desmoplakin, *DSP*, left-ventricular non-compaction cardiomyopathy, cardiovascular genetics, dilated cardiomyopathy

## Abstract

Here, we present a small Russian family, where the index patient received a diagnosis of left-ventricular non-compaction cardiomyopathy (LVNC) in combination with a skeletal myopathy. Clinical follow-up analysis revealed a LVNC phenotype also in her son. Therefore, we applied a broad next-generation sequencing gene panel approach for the identification of the underlying mutation. Interestingly, *DES*-p.A337P was identified in the genomes of both patients, whereas only the index patient carried *DSP*-p.L1348X. *DES* encodes the muscle-specific intermediate filament protein desmin and *DSP* encodes desmoplakin, which is a cytolinker protein connecting desmosomes with the intermediate filaments. Because the majority of *DES* mutations cause severe filament assembly defects and because this mutation was found in both affected patients, we analyzed this *DES* mutation in vitro by cell transfection experiments in combination with confocal microscopy. Of note, desmin-p.A337P forms cytoplasmic aggregates in transfected SW-13 cells and in cardiomyocytes derived from induced pluripotent stem cells underlining its pathogenicity. In conclusion, we suggest including the *DES* gene in the genetic analysis for LVNC patients in the future, especially if clinical involvement of the skeletal muscle is present.

## 1. Introduction

Left-ventricular non-compaction cardiomyopathy (LVNC) is a rare cardiomyopathy characterized by a pathological structure of the myocardium with a strongly thickened non-compacted layer in combination with hyper-trabeculation [1]. In recent years, LVNC has received increased interest from clinicians and basic scientists due to the improvements in imaging techniques and increased detection of LVNC [2]. There are about 80 genes associated with LVNC [1]. One of these genes is the *DES* gene encoding the muscle-specific intermediate filament protein desmin, which is expressed in all muscle cell types. Desmin filaments connect different cell organelles like the desmosomes, Z-bands, costameres, and nuclei with the cytoskeleton. More than 100 different pathogenic *DES* mutations have been described so far [3]. Most of these mutations are heterozygous, small in-frame deletion or missense mutations. Pathogenic *DES* mutations leading to exchanges against proline residues are frequent because its imino group is a potent breaker of α-helical structures. However, the phenotypes caused by *DES* mutations are diverse. They range from isolated skeletal myopathy to different cardiomyopathies, including dilated (DCM) [4], hypertrophic (HCM) [5], arrhythmogenic (ACM) [6,7,8], and restrictive cardiomyopathy (RCM) [9,10], or combinations of both [11]. However, only a few *DES* mutations are associated with LVNC [12,13,14,15]. At the molecular level, the majority of pathogenic *DES* mutations cause an abnormal desmin aggregation by interfering defects within the filament assembly process [16,17,18,19].

In this article, we present a small Russian family, where we identified a likely pathogenic heterozygous missense variant in *DES* (hg19:chr2:220285661, NM_001927.3:c.1009G>C, rs59962885), which caused the amino acid substitution p.A337P. Of note, mutation carriers presented LVNC in combination with skeletal myopathy. Previously, *DES*-p.A337P has been described in only one family with an adult-onset skeletal myopathy and mild cardiac involvement without indicating the presence of LVNC [20]. Moreover, we discuss the clinical significance of an additional likely pathogenic heterozygous variant in *DSP* (hg19:chr6:7580464, NM_004415.3:c.4042delC, p.L1348X), which was detected in one family member with LVNC and severe heart failure.

## 2. Materials and Methods

### 2.1. Clinical Description of the Patients

Two generations of the family with LVNC are presented. Family members underwent clinical examination, which included blood sample collection, biochemical and general examination, electrocardiography using 24 h Holter monitoring of electrocardiogram (HM-ECG), cardiac magnetic resonance imaging (MRI), and contrast echocardiography. Imaging echocardiography criteria were applied as previously suggested (criteria by Jenni and MRI criteria by Petersen [2,21]). This study was performed in accordance with the Declaration of Helsinki and was approved by the Institutional Review Boards of the National Research Center for Therapy and Preventive Medicine (Moscow, Russia), with written informed consent obtained from each participant and/or their legal representative, as appropriate.

### 2.2. Cardiac Magnetic Resonance Imaging

Cardiac MRI was performed with a 1.5-T imager (Avanto, Siemens Medical Solutions, Erlangen, Germany) using a standard protocol. Breath-hold cine MRI was performed using retrospective ECG-gated segmented true fast imaging with steady-state free-precession (SSFP). Cine MRI were acquired in long-axis and short-axis planes covering the whole left ventricle (LV) and right ventricle (RV). Late gadolinium enhancement (LGE) images were acquired in the same planes 15 min after intravenous injection of the gadolinium contrast agent (Gd-DTPA-BMA, Omniscan, WI, USA) in a dose of 0.15 mmol/kg using inversion-recovery turbo fast low-angle shot (FLASH) pulse sequence.

### 2.3. Molecular Genetic Analysis

DNA was isolated using the QIAamp^®^ DNA Blood Mini Kit (Qiagen, Hilden, Germany). DNA concentration was determined on a Qubit 4.0 fluorometer (Thermo Fisher Scientific, Waltham, MA, USA). Next-generation sequencing (NGS) was carried out on Ion S5 (Thermo Fisher Scientific, Waltham, MA, USA). Ampliseq libraries were prepared on Ion Chef System (Thermo Fisher Scientific, Waltham, MA, USA) using a custom panel developed in the Ion AmpliSeq Designer (Thermo Fisher Scientific, Waltham, MA, USA). The panel included exon sequences of 137 genes associated with LVNC or other cardiomyopathies (see Appendix A for a complete gene list). All stages of sequencing were carried out according to the manufacturer’s protocols. Sequencing and bioinformatics analysis resulted in FASTQ and VCF files. For clinical interpretation, genetic variants with frequencies in the gnomAD database of <1% were selected (https://gnomad.broadinstitute.org/) [22]. Evaluation of the pathogenicity of the variants was carried out in accordance with the recommendations of the American College of Medical Genetics and Genomics (ACMG) [23]. For verification by Sanger sequencing, the following oligonucleotides were used: 5′‑CTTGCTCATCCCTACCCGTG-3′ and 5′-ATGCCCAGGAACCCCTGATT-3′ (PCR product size—371 bp) for *DES* and 5′-AGGCCTGTGGCTCTGAGATA-3′ and 5′-GCTCCTGTTTTCTCGGGTGA-3′ (PCR product size—380 bp) for *DSP*. PCRs were performed in 20 μL of a mixture containing 0.2 mM of each nucleotide, 1x PCR buffer, 20 ng of the DNA, 10 ng of each primer, and 2.5 U of DNA polymerase. Amplifications were performed on a GeneAmp PCR System 9700 thermocycler (Thermo Fisher Scientific, Waltham, MA, USA) with the following parameters: 95 °C—300 s, 30 cycles: 95 °C—30 s, 62 °C—30 s, 72 °C—30 s, 72 °C—600 s. Before the Sanger reaction, the obtained amplicons were purified using ExoSAP-IT (Affymetrix, Santa Clara, CA, USA) according to the manufacturer’s protocol. The nucleotide sequence of PCR products was determined using the ABI PRISM^®^ BigDye ™ Terminator reagent kit v. 3.1 followed by analysis of the reaction products on an automated DNA sequencer Applied Biosystem 3500 DNA Analyzer (Thermo Fisher Scientific, Waltham, MA, USA).

### 2.4. Cloning, Site-Directed Mutagenesis, Cell Culture, and Confocal Microscopy

The plasmid pmRuby-N1-DES and pEYFP-N1-DES were previously described [24]. The QuikChange Lightning Site-Directed Mutagenesis kit (Agilent Technologies, Santa Clara, CA, USA) was used according to the manufacturer’s instructions to insert p.A337P. The *DES* cDNAs of all plasmids were verified by Sanger sequencing (Macrogen, Amsterdam, The Netherlands). PureLink HiPure Plasmid Midiprep kit (Thermo Fisher Scientific, Waltham, MA, USA) was used for plasmid preparation. SW‑13 cells, which do not express any endogenous cytoplasmic intermediate filament proteins, were cultured in Dulbecco’s Modified Eagle’s Medium (DMEM) supplemented with 10% fetal calf serum and Penicillin/Streptomycin (Thermo Fisher Scientific, Waltham, MA, USA) at 37 °C and 5% CO_2_. One day before transfection, the cells were trypsinized and cultured in µSlide 2-well chambers (ibidi GmbH, Martinsried, Germany). Lipofectamin 3000 (Thermo Fisher Scientific, Waltham, MA, USA) was used for cell transfection according to the manufacturer’s recommendations. Twenty-four hours after transfection, the cells were gently washed with phosphate buffered saline (PBS) and were fixed for 10 min at room temperature (RT) using 4% Histofix (Carl Roth, Karlsruhe, Germany). Afterwards, the cells were washed twice with PBS and were permeabilized using 0.1% Triton-X100 (10 min, RT). Phalloidin conjugated with Alexa488 and 4′,6-diamidin-2-phenylindol (DAPI) were used to stain F-actin and the nuclei. Confocal microscopy was performed as previously described using the TCS SP8 system (Leica Microsystems, Wetzlar, Germany) [25].

Human-induced pluripotent stem cells (hiPSC; NP00040–8, #UKKi011-A, European Bank for induced pluripotent Stem Cells) were kindly provided by Dr. Tomo Saric (University of Cologne, Cologne, Germany). HiPSCs were cultured in E8 medium (Thermo Fisher Scientific, Waltham, MA, USA) on vitronectin-coated cell culture plates and the medium was changed every day. Differentiation into cardiomyocytes was performed by modulation of the Wnt-pathway, as previously described [26]. The 4D Nuclefector system (Lonza, Cologne, Germany) was used for electroporation of hiPSC-derived cardiomyocytes using the P3 Primary Cell 4D-Nucleofector Kit and program CA-137. Afterwards, the cells were cultured in µ-Slide 2-well chambers (ibidi GmbH, Martinsried, Germany) for 24 h at 37 °C and 5% CO_2_. The cells were gently washed twice with PBS and were fixed with 4% Roti Histofix (Carl Roth, Karlsruhe, Germany) for 15 min at RT. 0.1% Triton X100 was used for plasma membrane permeabilization (15 min, RT). Anti α-actinin antibodies (Sigma-Aldrich, St Louis, MO, USA) were used in combination with secondary antibodies conjugated to Cy3 (Jackson Immuno Research, Newmarket, UK) for co-staining of cardiomyocytes. Nuclei were stained using 4′-6-diamidino-2-phenylindole (DAPI) (1 µg/mL) for 5 min at RT and were washed twice with PBS. Confocal microscopy has been done as previously described [9].

### 2.5. Statistical Analysis of Aggregate Formation in Transfected SW-13 Cells

About 100 cells per independent transfection experiment (*n* = 4) were analyzed by counting the number of aggregate-forming cells. The non-parametric Mann–Whitney test was performed using GraphPad Prism 8.3 (GraphPad Software, San Diego, CA, USA). *p*-values ≤ 0.05 were considered as significant.

## 3. Results

### 3.1. Clinical Investigations

**Figure 1:**
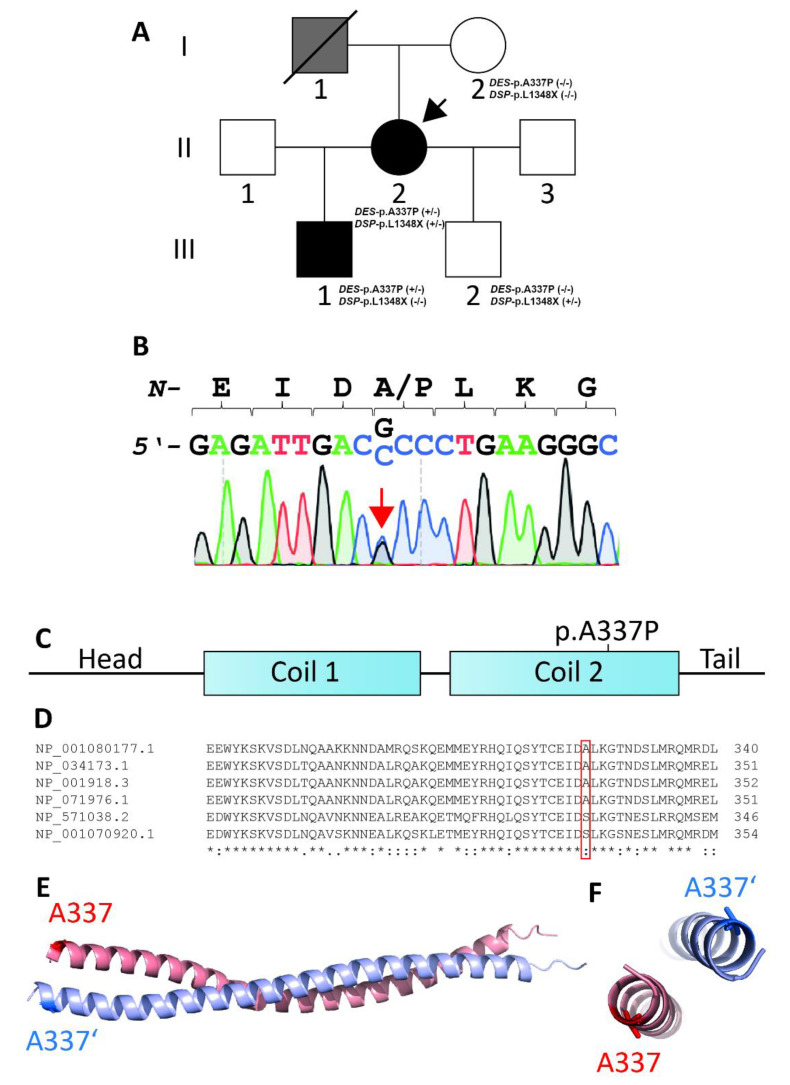
(**A**) Pedigree of the family. Circles represent females, squares males, slash denotes deceased. Black-filled symbols indicate a cardiac phenotype, grey-filled symbols indicate individuals with suspected cardiomyopathy, and white symbols indicate healthy family members. +/− or −/− indicates heterozygous or wild-type *DES*-p.A337P and *DSP*-p.L1348X variants, respectively. The index patient (II-2) is marked with an arrow. (**B**) Electropherograms of *DES*-p.A337P (c.1009G>C) in the genome of the index patient (II-2) and her son (III-1). (**C**) Schematic domain structure of desmin. *DES*-p.A337P is localized in the coil-2 sub-domain. (**D**) Multiple species alignment of desmin from *Xenopus laevis* (NP_001080177.1), *Mus musculus* (NP_034173.1), *Homo sapiens* (NP_001918.3), *Rattus norvegicus* (NP_071976.1), and *Danio rerio* (NP_571038.2, NP_001070920.1). (**E**,**F**) Desmin dimer, modeled using Swiss-Model (PDB ID: 3TRT [27]). Mutant alanine residues (p.A337 and p.A337′) are highlighted in blue and red. Of note, both alanine residues are localized at the opposite sites of the desmin dimer.

The index patient II-2 (Figure 1A) noted at the age of 25, during pregnancy, an episode of syncope and in the following years, developed stress-related arrhythmia.

**Figure 2:**
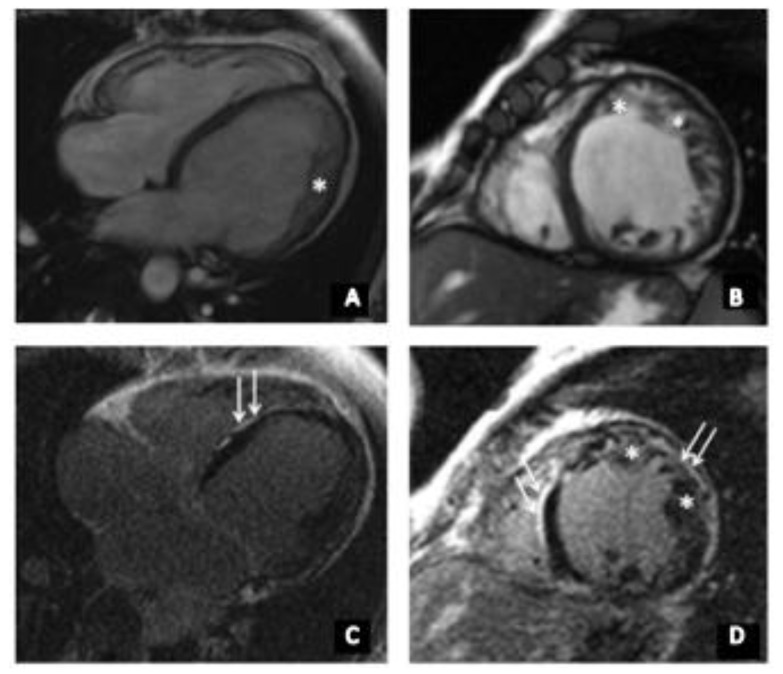
(**A**–**D**) cardiac MRI of index patient (II-2). * indicates non-compact myocardium (up to 18 mm). (**A**) Long-axis four-chamber projection, steady state free prcession (SSFP), and (**B**) short axis, SSFP, (**C**,**D**) Late gadolinium enhancement, areas of non-ischemic (subepicardial) contrast enhancement of left-ventricular walls are indicated with the white arrows.

At the age of 35, she had acute respiratory viral infection (ARVI), lasting for about a month with febrile temperature and severe asthenic syndrome. Afterwards, she began to note the worsening of heart failure, shortness of breath when climbing to the second floor, and a decrease in exercise tolerance. ECG detected a left bundle branch block (LBBB). Echocardiography revealed an ejection fraction (EF) of 42% and the left-ventricular end diastolic diameter (LVED) was 6.3 cm. In addition, left-ventricular (LV) diffuse hypokinesis was found. HM-ECG showed a sinus rhythm with heart rate of 34–151, frequent premature supraventricular beats (PSVB), and premature ventricular beats (PVB). She received β-blocker therapy and an angiotensin-converting enzyme inhibitor, which the patient independently canceled due to poor health. At the age of 36, she was admitted to the National Medical Research Center for Therapy and Preventive Medicine (Moscow, Russia). Her expressed muscle weakness while climbing the stairs attracted our clinical attention. According to echocardiography, the signs of a non-compact LV myocardium were detected in the apex, lateral, anterior, and posterior walls with the formation of non-compacted cardiomyopathy. It is impossible to exclude transferred myocarditis, with a decrease in LV systolic function up to 28%. To clarify the diagnosis, the patient underwent cardiac MRI with gadolinium (Figure 2), which showed a two-layer structure of the LV myocardium in the lower, anterior, anterolateral, and lower lateral segments, corresponding to the criteria for LVNC (in more than six segments). Thickness of the compact layer in these segments was 1.8–3.4 mm, and non-compacted was 10–17 mm. The non-compacted layer was presented by a spongy myocardium. The indexed mass of a non-compacted myocardium was 41 g/m^2^ (at a rate of up to 15 g/m^2^), which was 37.7% of the mass of the LV myocardium (at a rate of up to 25%), and ejection fraction (EF) was 31%. Tests for cardiotropic viruses were negative. According to the HM-ECG, non-sustained ventricular tachycardia (NSVT) was detected, and the patient received amiodarone for treatment of arrhythmia. Electroneuromyography revealed a muscle lesion level with mild signs of the ongoing breakdown of muscle fibers in the thigh muscle. At the age of 37, a cardiac resynchronization therapy device (CRT‑d) was implanted due to the presence of heart failure, reduced EF, and the presence of LBBB (QRS > 160 m/s). The patient’s condition remained stable, in view of which she independently reduced her therapy. Thus, instead of a decrease in EF to 23%, an increase in pulmonary systolic pressure (PSP) was noted during a planned examination in 2020. Currently, the patient constantly receives bisoprolol 2.5 mg, perindopril 2.5 mg, torasemide 5 mg, spironolactone 25 mg, and warfarin 5 mg. The father of the index patient (Figure 1A, I-1) died at the age of 52. Although no detailed clinical data are available, a cardiomyopathy can be suggested. At the age of 16, the first son of the index patient (Figure 1A, III-1) developed muscle weakness and an unexpressed shortness of breath with significant exertion. At the age of 18, an ambulatory examination was carried out with respect to the identified disease of the mother. Echocardiography showed signs of non-compacted myocardium (Chin criteria). The heart chambers were not expanded. Diffuse hypokinesis was found. HM-ECG showed episodes of sinoatrial (SA) blockade of the second degree. Cardiac MRI with gadolinium suggested LVNC (Figure 2). There were no signs of myocarditis.

The second son (Figure 1A, III-2), 17 years old, underwent a comprehensive cardiological and neurological examination. According to the results of echocardiography and MRI, signs of non-compacted myocardium and myopathy were absent.

### 3.2. Genetic and Functional Analysis

NGS sequencing using a gene panel covering the most likely cardiomyopathy genes was carried out for the index patient (II-2) and her son (III-1). In the index patient, 686 sequence variants were identified. Of these, only 6 variants with minor allele frequency (MAF) < 1% and influence on the amino acid sequence of genes were identified (nonsense, missense, and short indels) (Table 1). No splicing variants were identified. We identified heterozygous non-synonymous missense variant *DES*-p.A337P (c.1009G>C) in exon-6 of the *DES* gene in the genome of both affected patients (hg19:chr2:220285661, NM_001927.3, rs59962885) and verified it using Sanger sequencing (Figure 1B). This variant is located in the highly conserved 2B helical segment of desmin (Figure 1C–E) and causes enhanced adhesiveness, which leads to filament aggregation, as previously described [20]. *DES*-p.A337P is not listed in the Genome Aggregation Database (https://gnomad.broadinstitute.org/, December 2020) [22]. Several bioinformatic prediction tools suggested a high probability for a damaging effect of *DES*-p.A337P (Table 2). Based on the ACMG criteria, this variant had a category of unknown significance (PM2. PP1, PP3, PP5), but after the functional analysis, the category changed to likely pathogenic (PS3, PM2. PP1, PP3, PP5). Another putative heterozygous likely pathogenic nonsense *DSP* variant was found only in the genome of patient II-2 (hg19:chr6:7580464, NM_004415.3: c.4042delC), which led to a premature termination codon at position 1384 (p.L1384X). *DSP* encodes desmoplakin, which is a direct binding partner of desmin and which mediates the molecular linkage of intermediate filaments with the cardiac desmosomes. Sanger sequencing of *DES* and *DSP* mutations was done not only for II-2 and III-1, but also for I-2 and III-2. The *DES*-p.A337P variant was found in II-2 and III-1, the *DSP*-p.L1348X variant in II-2 and III-2.

**Figure 3:**
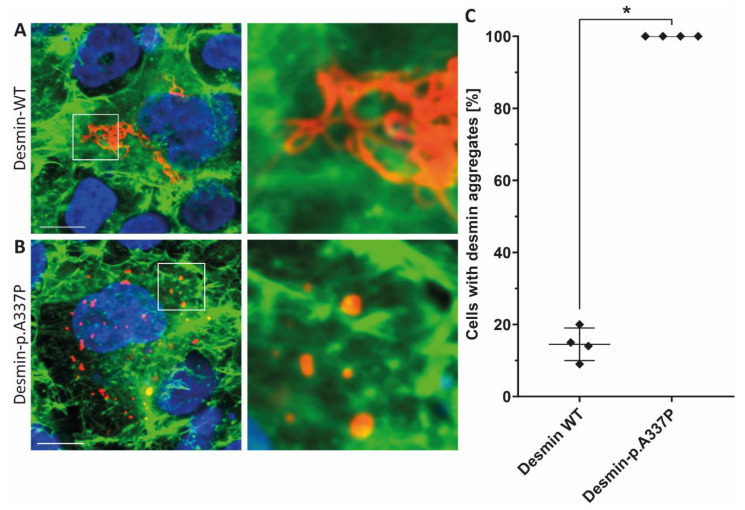
Representative confocal microscopy images of SW-13 cells expressing wild-type desmin (**A**) or desmin-p.A337P (**B**) (shown in red). The fluorescence intensity of Alexa-488 conjugated to phalloidin (for F-actin staining) is shown in green and the nuclei were stained using DAPI (blue). Scale bars represent 10 µm. (**C**) Statistical analysis of desmin aggregate formation revealed for desmin-p.A337P an abnormal cytoplasmic aggregate formation in the majority of transfected SW-13 cells. * *p*-value < 0.05.

**Figure 4:**
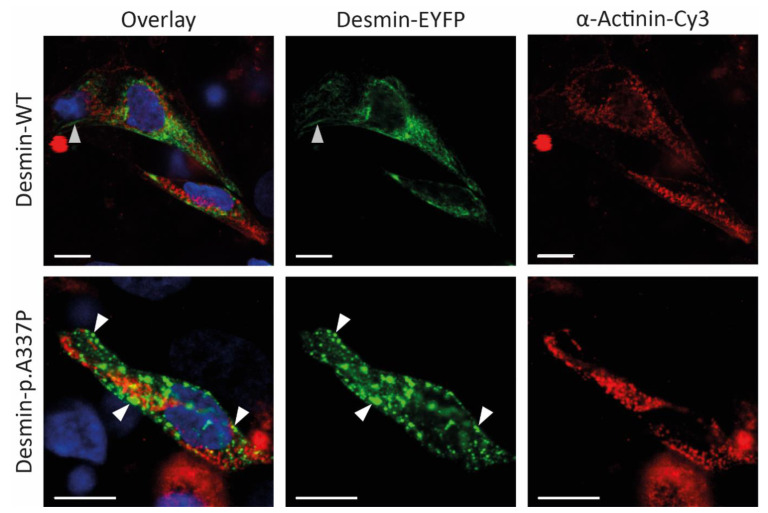
Representative confocal microscopy images of iPSC-derived cardiomyocytes expressing wild-type or mutant desmin (shown in green). The cardiomyocyte marker α-actinin is shown in red and nuclei are shown in blue. Of note, mutant desmin forms cytoplasmic aggregates (white arrows), whereas wild-type desmin forms filamentous structures (grey arrows). Scale bars represent 10 µm.

Because an abnormal desmin aggregation is typical for *DES* mutations, we expressed this mutation in SW-13 cells (Figure 3) and in hiPSC-derived cardiomyocytes (Figure 4). SW-13 cells were used because they do not express any cytoplasmic endogenous intermediate filament proteins [34].

In contrast, hiPSC-derived cardiomyocytes express endogenous desmin. Confocal microscopy revealed, in the majority of transfected cells, an abnormal desmin aggregation for both cell types, whereas the wild-type desmin forms regular intermediate filaments (Figure 3 and Figure 4). In conclusion, these transfection experiments support a pathogenic effect of *DES*-p.A337P.

In summary, our clinical, genetic, and functional data support that *DES*-p.A337P is the most likely cause for LVNC in combination with skeletal myopathy in the described family. Our findings expand the phenotypes associated with *DES* mutations and underline a broad clinical presentation of different *DES* mutation carriers. However, we cannot exclude that heterozygous *DSP*-p.L1348X might also contribute to the cardiac phenotype in the index patient II-2, although the majority of known pathogenic *DSP* mutations were recessively inherited.

## 4. Discussion

Mutations in the *DES* gene cause various types of skeletal myopathies or cardiomyopathies. Interestingly, the cardiac spectrum of different *DES*-associated cardiomyopathies is broad and includes DCM [24,35], ACM [6,7], and RCM [9,10]. In the last few years, the association of *DES* mutations with LVNC has been shown. In the study by Miszalski-Jamka et al., 3 of 174 patients had a mutation in the *DES* gene (p.A360S, p.L69P, p.R212Q), but the pathogenicity of these variants was not investigated [14]. Recently, we described a three-generation family having LVNC in combination with a skeletal myopathy leading to heart transplantation and sudden cardiac death. This family carried a small pathogenic in-frame deletion (p.Q113-L115del) in the *DES* gene [13]. A meta-analysis by Waning et al. revealed the main genes responsible for LVNC [1]. Mutations in the *DES* gene were assigned to a group of rare sarcomeric genes that accounted for 6% of the total number of all pathogenic mutations. Despite the rare occurrence of mutations in this gene associated with LVNC, patients with neuromuscular and heart diseases tend to have a poorer prognosis and less effective standard treatment [36,37].

Here, we present an additional family with *DES* mutation where LVNC was diagnosed in the index patient (II-2) and her son (III-1). The index patient belonged to a dilated type with reduced systolic function, severe heart failure, heart rhythm and conduction disorders (paroxysmal atrial fibrillation, NSVT, and LBBB), and isolated LVNC with preserved LV, without clinical manifestations of cardiomyopathy in her son. Also, there are manifestations of myopathy in the proband’s son (muscle weakness), asthenic physique, which are characteristic of desminopathy. In a study by van Spaendonck-Zwarts et al., it was shown that most pathogenic *DES* mutations associated with myopathies reside within either the 2B helical segment or the tail [38]. In our case, we see a similar clinical presentation consisting of combined neurological and cardiac symptoms in the presence of a rare *DES* missense mutation (p.A337P), which is also localized in helical coil-2 segment of the rod domain. The majority of pathogenic *DES* mutations are missense mutations leading to severe filament assembly defects [16,17]. Therefore, we performed transfection experiments of SW-13- and iPSC-derived cardiomyocytes, revealing a severe filament assembly defect of mutant desmin-p.A337P. These results are in good agreement with filament assembly experiments of the recombinant purified desmin-p.A337P analyzed by transmission electron microscopy [16]. Together, these data underline the pathogenic impact of this desmin mutation.

In addition to the *DES* mutation p.A337P, we found a novel nonsense variant *DSP*-p.L1348X in the genome of II-2 but not in her son (III-1). It could be suggested that the clinical differences between phenotypes within this described family might be due to a combination of pathological mutations in the *DES* and *DSP* genes, which might also explain the more clinical severity of the index patient in comparison to her son. The *DSP* gene encodes desmoplakin, which is a structural linker protein of desmosomes in cardiomyocytes and epidermal cells. Desmoplakin links the desmosomes with the intermediate filaments, which are formed in cardiomyocytes by desmin [3]. The majority of pathogenic *DSP* mutations were frequently found as homozygous or compound heterozygous mutations in patients presenting arrhythmogenic right ventricular cardiomyopathy (ARVC) in combination with wooly hair and palmoplantar keratoderma [39,40]. This triad of clinical symptoms is known as Carvajal syndrome (OMIM #605676). However, some publications describe heterozygous *DSP* mutations associated with DCM, e.g., Lopez et al. showed that from 24 patients with ARVC and 23 with DCM, 3 patients carried novel *DSP* variants. Cascade family screening led to the identification of 15 relatives who were variant carriers. Interestingly, cardiac magnetic resonance revealed a non-compaction phenotype in five of them [41].

One limitation of our study is that not all relatives were available for clinical and genetic screening (the affected father of the proband died earlier), and that copy number variants were not investigated.

## 5. Conclusions

In conclusion, we presented a family with LVNC carrying a likely pathogenic *DES* missense variant (p.A337P). Mutant desmin-p.A337P forms abnormal cytoplasmic aggregates. In addition, we suggested that additional likely pathogenic variants like *DSP*-p.L1348X in the described index patient might contribute to the clinical severity. Therefore, *DES* and presumably desmosomal genes like *DSP* should be genetically investigated in LVNC patients, especially if the skeletal muscle is also affected.

## Figures and Tables

**Table 1:** List of the rare variants (MAF < 1%) identified in the proband and her relatives.

Gene	Genomic Coordinates	Transcript; DNA Change; Protein Change	dbSNP ID	gnomAD MAF (v. 2.1.1)	ACMG Interpretation	Familial Aggregation with LVNC	The Presence of the Variants in A Family Member			
							I-2	II-2	III-1	III-2
*DSP*	hg19:chr6:7580464_AC/A	NM_004415.3; c.4042delC; p.Leu1348Ter	-	-	likely pathogenic(PVS1, PM2, BS4)	no	0/0	1/0	0/0	1/0
*DES*	hg19:chr2:220285661_G/C	NM_001927.3; c.1009G>C; p.Ala337Pro	rs59962885	-	likely pathogenic(PS3, PM2. PP1, PP3, PP5)	yes	0/0	1/0	1/0	0/0
*PKP2*	hg19:chr12:32994058_A/C	NM_004572.3; c.1592T>G; p.Ile531Ser	rs147240502	0.004746	likely benign (PM1, PP1, BS1, BP6)	yes	N/A	1/0	1/0	n/a
*MYH7*	hg19:chr20:33581180_G/A	NM_020884.4; c.2477G>A; p.Arg826His	rs187028260	0.002979	Benign (PM1, PP3, BS1, BS2)	no	N/A	1/0	0/0	n/a
*TTN*	hg19:chr2:179395554_GC/AA	NM_001256850.1; c.100864_100865delGCinsTT; p.Ala33622Phe	rs794729250	-	likely benign (PM2, PP3, BS4, BP1, BP6)	no	N/A	1/0	0/0	n/a
*DES*	hg19:chr2:220284876_C/T	NM_001927.3; c.638C>T; p.Ala213Val	rs41272699	0.009280	likely benign (PP1, PP3, BS1, BP6)	yes	N/A	1/0	1/0	n/a

MAF—minor allele frequency. LVNC—left- ventricular non-compaction. N/A—not applicable.

**Table 2:** Overview of different prediction tools analyzing *DES*-p.A337P.

PolyPhen-2 [28]	Mutation Taster [29]	PROVEAN [30]	PANTHER [31]	SNPs&GO [32]	SIFT [33]
Probably damaging 0.991	Disease-causing 27	Deleterious −3.293	Probably damaging 455	Disease-associated variant 0.758	Deleterious

## Data Availability

The datasets used and/or analyzed during the current study are available from the corresponding author on reasonable request.

## Appendix A

Genes included in the cardiomyopathy NGS panel: *ABCC9*, *ACADVL*, *ACTA1*, *ACTC1*, *ACTN2*, *AGK*, *AGL*, *AGPAT2*, *ALMS1*, *AMPD1*, *ANK2*, *ANKRD1*, *BAG3*, *CACNA1C*, *CALR3*, *CASQ2*, *CAV3*, *COQ2*, *COX15*, *COX6B1*, *CRYAB*, *CSRP3*, *CTF1*, *CTNNA3*, *DES*, *DMD*, *DMPK*, *DNAJC19*, *DNM1L*, *DOLK*, *DSC2*, *DSG2*, *DSP*, *DTNA*, *EMD*, *EYA4*, *FBN2*, *FHL1*, *FHL2*, *FHOD3*, *FKTN*, *FLNC*, *FOXD4*, *GATA4*, *GATA6*, *GATAD1*, *GJA5*, *GLA*, *GLB1*, *HADHB*, *HCCS*, *HCN4*, *HFE*, *HRAS*, *ILK*, *JPH2*, *JUP*, *KCNE1*, *KCNE2*, *KCNH2*, *KCNJ2*, *KCNJ8*, *KCNQ1*, *LAMA4*, *LAMP2*, *LDB3*, *LMNA*, *MEF2A*, *MIB1*, *MRPL3*, *MRPS22*, *MTO1*, *MURC*, *MYBPC3*, *MYH6*, *MYH7*, *MYH7B*, *MYL2*, *MYL3*, *MYLK2*, *MYOM1*, *MYOT*, *MYOZ2*, *MYPN*, *NEBL*, *NEXN*, *NKX2-5*, *NNT*, *NOTCH1*, *PDLIM3*, *PHKA1*, *PKP2*, *PLEC*, *PLEKHM2*, *PLN*, *PMP22*, *PRDM16*, *PRKAG2*, *PSEN1*, *PSEN2*, *PTPN11*, *RAF1*, *RBM20*, *RYR2*, *SCN1B*, *SCN5A*, *SCO2*, *SDHA*, *SDHD*, *SGCD*, *SHOC2*, *SIX1*, *SLC25A3*, *SLC25A4*, *SURF1*, *SYNE1*, *SYNE2*, *TAZ*, *TBX1*, *TBX20*, *TBX5*, *TCAP*, *TGFB3*, *TMEM43*, *TMEM70*, *TMPO*, *TNNC1*, *TNNI3*, *TNNT2*, *TNNT3*, *TPM1*, *TRIM63*, *TSFM*, *TTN*, *TTR*, *TXNRD2*, *VCL*.
